# Outpatient parenteral antimicrobial therapy (OPAT) in Germany: insights and clinical outcomes from the K-APAT cohort study

**DOI:** 10.1007/s15010-024-02199-9

**Published:** 2024-03-13

**Authors:** Kirsten Schmidt-Hellerau, Nina Baade, Marina Günther, Nadine Scholten, Christoph Heinrich Lindemann, Charlotte Leisse, Charlotte Oberröhrmann, Sophie Peter, Norma Jung, Isabelle Suarez, Carola Horn, Peter Ihle, Jutta Küpper-Nybelen, Anna Hagemeier, Martin Hellmich, Clara Lehmann

**Affiliations:** 1 Department I of Internal Medicine, University of Cologne, Faculty of Medicine and University Hospital Cologne, Cologne, Germany; 2 Institute of Medical Sociology, Health Services Research and Rehabilitation Science, Chair of Health Services Research, University of Cologne, Faculty of Medicine and University Hospital Cologne, Cologne, Germany; 3 Department II of Internal Medicine and Center for Molecular Medicine Cologne (CMMC), University of Cologne, Faculty of Medicine and University Hospital Cologne, Cologne, Germany; 4 Chair of General Practice II and Patient-Centeredness in Primary Care, Institute of General Practice and Primary Care, Faculty of Health, Witten/Herdecke University, Witten, Germany; 5 PMV Forschungsgruppe, Faculty of Medicine and University Hospital Cologne, Cologne, Germany; 6 Institute of Medical Statistics and Computational Biology (IMSB), Faculty of Medicine and University Hospital Cologne, University of Cologne, Cologne, Germany; 7 Center for Molecular Medicine Cologne (CMMC), University of Cologne, Cologne, Germany; 8 German Center for Infection Research (DZIF), Bonn-Cologne, Germany

**Keywords:** Outpatient parenteral antimicrobial Therapy (OPAT), Infectious Diseases, Outpatient Sector, Antibiotics, Antimicrobials

## Abstract

**Purpose:**

Outpatient parenteral antimicrobial therapy (OPAT) offers several key advantages, including enhanced patient quality of life, reduced healthcare costs, and a potential reduction of nosocomial infections. It is acknowledged for its safety and effectiveness. This study provides the first systematic clinical data for Germany, where OPAT has not yet been widely adopted. The aim is to establish a foundational reference point for further research and integration of OPAT into the German healthcare system.

**Methods:**

This prospective observational study descriptively analyses data obtained from a cohort of patients receiving OPAT. Both in- and outpatients from all medical specialties could be recruited. Patients administered the anti-infective medications themselves at home using elastomeric pumps.

**Results:**

77 patients received OPAT, with a median duration of 15 days and saving 1782 inpatient days. The most frequently treated entities were orthopaedic infections (*n* = 20, 26%), *S. aureus* bloodstream infection (*n* = 16, 21%) and infectious endocarditis (*n* = 11, 14%). The most frequently applied drugs were flucloxacillin (*n* = 18, 23%), penicillin G (*n* = 13, 17%) and ceftriaxone (*n* = 10; 13%). Only 5% of patients (*n* = 4) reported to have missed more than one outpatient dose (max. 3 per patient). Only one catheter-related adverse event required medical intervention, and there were no catheter-related infections.

**Conclusion:**

The study demonstrates that OPAT can be safely conducted in Germany. In preparation for its broader implementation, crucial next steps include creating medical guidelines, fostering interdisciplinary and inter-sectoral communication, as well as creating financial and structural regulations that facilitate and encourage the adoption of OPAT.

**Trial registration number:**

NCT04002453.

**Supplementary Information:**

The online version contains supplementary material available at 10.1007/s15010-024-02199-9.

## Introduction

Outpatient parenteral antimicrobial therapy (OPAT) has become an integral part of healthcare delivery in several countries [1, 2]. Key advantages of OPAT include shorter or avoided hospital stays, leading to a decrease in nosocomial infections and costs, while simultaneously increasing patients’ quality of life [1, 3–8]. Challenges include complications generally related to any intravenous (IV) treatment (such as local thrombosis, local infection and extravasation), but also related to its outpatient administration (e.g., less frequent clinical assessment of the patient, and in case of self-administration potentially incorrect handling of materials). Potential pitfalls of OPAT as a mode of treatment have been assumed to include unnecessary long treatment durations, unnecessary use of IV drugs instead of an oral alternative, unnecessary use of broad-spectrum antibiotics because of more favourable dosing intervals, and failing to perform surgical interventions [2, 9]. The latter have led to the recommendation of interdisciplinary management involving infectious disease (ID) specialist assessment [1]. Selection criteria to identify patients suitable for OPAT should include medical prerequisites (e.g., controlled infection, stable clinical condition and comorbidities at the time of discharge) as well as cognitive and social factors and patient adherence [1, 2, 10].

OPAT can be administered through various methods, including engagement of a mobile nursing service, self-administration using mechanical pumps at home, or visits to a designated outpatient facility [10, 11]. However, there is little data available regarding differences in efficacy [1]. An appropriate IV catheter is a prerequisite. Peripheral lines often necessitate frequent renewals, making alternatives such as peripherally inserted central catheters (PICC lines) or midlines more convenient. In some cases, previously inserted catheters like port catheters may be used. Regular clinical and laboratory treatment monitoring is crucial [1] and is commonly ensured by regular outpatient visits at specialized centres. Typically, an ID physician assumes overall responsibility, and recommended dedicated OPAT teams consist of a lead OPAT nurse, an antimicrobial pharmacist and an ID specialist [2].

Despite the advantages of OPAT and its successful implementation elsewhere, it has yet to gain widespread adoption in Germany. Currently, national treatment recommendations and regulations are lacking. Existing data on OPAT in Germany is scarce, mainly retrospective and restricted to specific patient groups [12, 13]. The primary objective of this prospective observational study is to provide a first description of a cohort of diverse patients receiving OPAT in Germany, drawing on data from the K-APAT cohort (“outpatient parenteral antimicrobial treatment in the metropolitan region of Cologne”). This study aims to demonstrate the need for, the feasibility and acceptability of OPAT in Germany, while also characterizing the patient population and the types of infections treated. These first insights serve as a foundational reference point for further research and future implementation of OPAT in the German healthcare system.

## Methods

### Sample size

Based on a prospective screening in 2017 and calculating a rejection rate and a dropout rate of 15% each, a sample size of 120 patients over the course of 2 years was estimated to be feasible. This number is sufficient to estimate proportions with a precision below ± 5% (standard error).

### Patient population and study design

**Fig. 1:**
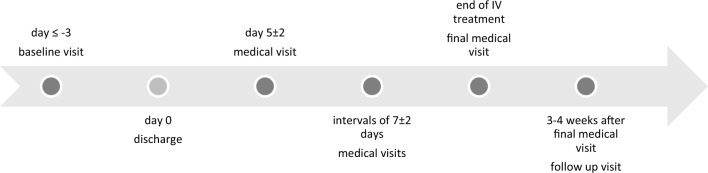
Timing of study visits

The study protocol of this prospective observational study has been published previously [14]. Between September 2019 and September 2021, patients suitable for OPAT according to international guidelines [1, 2] were included. Participants could be recruited from all departments of Cologne University Hospital and from four non-university hospitals and five physicians’ offices that are part of the Cologne Network of Infectious Diseases. Requirements for participation were a documented infection requiring IV treatment for at least 5 remaining days, the ability to provide written informed consent, clinical stability without conditions requiring inpatient treatment, and physical and mental suitability for OPAT. Indications for OPAT and individual patient suitability were assessed by an ID specialist using a checklist (supplementary material). The choice of anti-infective treatment followed international recommendations and current medical knowledge. Patients self-applied the anti-infective substances at home using mechanical (elastomeric) pumps. Each of the portable elastomeric pumps contains one dose, which is, after connection of the pump to the IV line, delivered with a defined infusion rate. Once the full dose is applied, the patient disconnects the pump. Before initiation of OPAT, patients and, if necessary, their caretakers were trained in handling the materials including management of the IV access, connection and disconnection of the pumps and storage of materials. The first dose was then applied by the patients under supervision. They received contact information of the pharmaceutical provider involved in their care, of the ID outpatient department and emergency department, and were instructed to call and/or present in case of any technical questions or any deterioration of symptoms. The strictly technical aspects, such as patient training, preparation and home delivery of drugs and materials, as well as weekly home visits to check on the IV access, were taken over by pharmaceutical providers. Medical responsibility remained with the ID department. See Fig. 1 for the timing of study visits.

### Data collection and statistical analyses

Sociodemographic and medical data as well as data on OPAT processes were collected by the treating physician from the ID outpatient department. Pseudonymized data from paper-based case report forms were transferred to a database using the online platform REDCap, and access was restricted to authorized study personnel. Data were analysed descriptively using Stata 16 and SAS 9.4. The analyses refer to all patients who started OPAT therapy (per protocol).

### Ethical considerations

The study has been approved by the Institutional Review Board of the University of Cologne, Germany (19–1284-1). Written informed consent was obtained from all participants.

## Results

Of 94 patients screened, 78 fulfilled the inclusion criteria and gave written consent. Among the reasons for exclusion were a lack of indication for IV treatment and clinical conditions impeding planned discharge. As one patient could not start OPAT after it had already been planned due to acute clinical deterioration, 77 participants were included in the analysis. The majority of participants were recruited at Cologne University hospital (*n* = 68, 88%), the remaining at other hospitals (*n* = 7, 9%) and outpatient offices (*n* = 2, 3%). Overall, including participants from Cologne University Hospital outpatient units, OPAT was initiated in seven outpatients (*n* = 7, 9%). Median age was 56 years (interquartile range = 40–66), and the oldest patient was 91 years old (range 19–91). See Table 1 for patient characteristics.

**Table 1:** Characteristics and pre-existing conditions of patients receiving OPAT (*n* = 77)

	*N* (%)
Age > 65 years	22 (29)
Female	23 (30)
Employed (incl. self-employed)	41 (53)
Single-person household	21 (27)
Any comorbidity	72 (94)
Cerebrovascular disease	6 (8)
Chronic heart failure	8 (10)
Chronic pulmonary disease	5 (6)
Diabetes mellitus	17 (22)
Gastroduodenal ulcer	1 (1)
Human immunodeficiency virus infection	7 (9)
Immunosuppression	21 (27)
Leukaemia	10 (13)
Lymphoma	7 (9)
Moderate or severe kidney disease	9 (12)
Moderate or severe liver disease	2 (3)
Myocardial infarction	5 (6)
Peripheral artery disease	2 (3)
Rheumatologic disease	6 (8)
Solid malignancy	8 (10)

Orthopaedic infections were treated in a quarter of patients (*n* = 20, 26%) (see Table 2). Every fifth patient was treated for *Staphylococcus aureus* bloodstream infection (*n* = 16, 21%). Secondary foci in the 11 patients with complicated *S. aureus* bloodstream infections were mostly orthopaedic (*n* = 5), followed by pulmonary and renal metastatic infection (each *n* = 2). Patients in which OPAT was initiated in an outpatient setting mainly suffered from neurosyphilis (5 of 7 patients). The majority of infections were monobacterial, see Table 3 for involved pathogens. In most cases, the indication for IV treatment was the type of infection, but in 22% of patients (*n* = 17) it was a lack of oral treatment options. Flucloxacillin (*n* = 18, 23%), penicillin G (*n* = 13, 17%) and ceftriaxone (*n* = 10; 13%) were administered most frequently and accounted for half of the substances administered (see Table 4). The indication for IV administration of ciprofloxacin and cotrimoxazole, substances that can in most cases be taken orally, was severe nocardiosis. Cotrimoxazole is one of the exceptions in which the stability of the substance does not allow for its use in elastomeric pumps, it was provided to the patients as a pre-prepared solution. Changes of treatment (other than a planned switch to sequential oral treatment) occurred in eight patients, mainly due to new microbiological findings (*n* = 3) and adverse events (*n* = 3).

**Table 2:** Infections treated by OPAT (multiple sites of infection per case possible) (*n* = 77)

	*N* (%)
Joint and bone infections	20 (26)
Vertebral osteomyelitis	11 (14)
Periprosthetic joint infection	7 (9)
Septic arthritis	2 (3)
*Staphylococcus aureus* bloodstream infection	16 (21)
uncomplicated	5 (6)
complicated	11 (14)
Infectious endocarditis	11 (14)
Neurosyphilis	9 (12)
Intracranial abscess	4 (5)
Renal abscess	4 (5)
Pulmonary infection	3 (4)
Pleural empyema	3 (4)
Others^1^	25 (32)

^1^2 each with soft tissue infections, urinary tract infection, hepatosplenic candidiasis, otitis externa maligna, cytomegalovirus, psoas abscess, vascular prosthesis infection; 1 each with nocardiosis, *Enterococcus faecium* bloodstream infection*,* disseminated *Streptococcus pyogenes* infection, chronic pansinusitis, multidrug-resistant pulmonary tuberculosis, exacerbation of cystic fibrosis, Lyme carditis, orbital and maxillary abscess with meningitis, oropharyngeal infection, invasive pulmonary aspergillosis, superinfected thrombosis

**Table 3:** Involved pathogens in patients receiving OPAT (*n* = 77)

	*N* (% of patients)
*Staphylococcus aureus*	21 (27)
*Staphylococcus epidermidis*	12 (16)
*Treponema pallidum*	9 (12)
*Viridans streptococci*	5 (7)
*Escherichia coli*	5 (7)
*Pseudomonas aeruginosa*	4 (5)
*Cutibacterium acnes*	4 (5)
Others^2^	16 (21)
No pathogen identified	4 (5)

Multiple pathogens per patient possible^1^

^1^Polymicrobial infections were treated in 4 patients (5%) (2 pathogens in each)

^2^In 2 patients each: *Cytomegalovirus, Fusobacterium, Nocardia;* 1 patient each: *Aeromonas caviae, Borrelia, Cardiobacterium hominis, Enterococcus faecalis, Enterococcus faecium, Haemophilus influenzae, Mycobacterium tuberculosis, Serratia marcescens, Streptococcus gallolyticus, Streptococcus pyogenes*

**Table 4:** Anti-infective drugs used for OPAT (*n* = 77), multiple drugs per patient were possible

	*N* (% of patients)
Flucloxacillin	18 (23)
Penicillin G	13 (17)
Ceftriaxone	10 (13)
Vancomycin	8 (10)
Meropenem	6 (8)
Cefazolin	4 (5)
Fosfomycin	4 (5)
Caspofungin	3 (4)
Others^2^	17 (22)

^1^6 patients each received 2 substances

^2^In 2 patients each: ampicillin/sulbactam, cotrimoxazole, daptomycin, ertapenem, ganciclovir, gentamicin; in 1 patient each: amikacin, ampicillin, ceftazidime, ciprofloxacin and piperacillin/tazobactam

Median duration of OPAT was 15 days (IQR 11–26, range 5–127 days). On average, 23 days of OPAT per patient resulted in 1782 accordingly saved hospital days. The medium number of OPAT clinical visits was four (IQR 3–5, range 3–18). Patients had been hospitalized for a median of 20 days before OPAT initiation (IQR 12–27; range 6–98). IV access was mainly via PICC lines (*n* = 66, 86%). In some cases, family members either helped with the drug administration (*n* = 7, 10%; missing data for *n* = 6) or took over completely (*n* = 5, 7%; missing data for *n* = 6). In one patient (1%), a mobile nursing service who had previously cared for the patient applied each dose. Only 5% (*n* = 4) of patients reported to have missed more than one outpatient dose (max. 3 doses per patient). Missed administrations were reported to be due to technical issues, catheter-related problems, patient forgetfulness and delayed drug delivery. Before completion of OPAT, 16 patients (21%) were rehospitalized, and further two during the follow-up period. Of the rehospitalizations during OPAT, 56% (*n* = 9) were directly related to the underlying disease: 25% were planned rehospitalizations (*n* = 5; 2 for surgery, 1 for rehabilitation, and 1 for diagnostic procedures) and 31% due to worsening of the underlying disease or complications of its treatment (*n* = 5). Another 25% (*n* = 4) were due to new conditions unrelated to the infection treated by OPAT, the anti-infectives used or OPAT itself. Of the three rehospitalizations neither due to the underlying condition nor to a new and clearly unrelated disease (19% of rehospitalizations resp. 4% of total), only one (6% resp. 1%), due to a (reversible) side effect of the antibiotic administered by OPAT, was associated with the infection treated by OPAT, the anti-infectives used or OPAT itself. The remaining two (13% resp. 3%) were admitted after an out-of-hospital cardiac arrest and after a fall, both without a diagnosed relation to the infection treated by OPAT and/or OPAT itself (both patients did not survive the hospital stay and are described in more detail below). Severe catheter-related adverse events (Common Terminology for Adverse Events (CTCAE) grade 3 and 4) were reported in 4 patients (5%): In 2 of 75 patients using central lines (3%), a new PICC line had to be inserted (due to clogging and accidental removal), one haematoma resolved spontaneously, and one PICC line had to be repositioned because its position had triggered cardiac arrhythmia. There were no diagnosed catheter-related infections. Not catheter-related adverse events were assessed to be related to the anti-infective drugs or the infection, not to their outpatient administration, and were mostly minor. Adverse events graded CTCAE 3 were registered in 3 patients (4%) (worsening chronic exertional dyspnoea, nausea in possible relation to Fosfomycin, and diarrhoea assessed to be due to graft-versus-host disease and cytomegaly virus reactivation), and one as CTCAE grade 4 (pacemaker pocket infection requiring surgical revision). Treatment changes due to minor adverse events were reported in 3 patients.

Clinical outcome was assessed at the end of the treatment visit (see Table 5). When including the follow-up period of 3–4 weeks after the end of OPAT, four patients (5%) had died overall. Two of them had had a previously known very limited life expectancy due to underlying late-stage malignancy. One patient, who was treated for invasive aspergillus infection after recent allogeneic stem cell transplantation, died from severe sepsis after rehospitalization after a fall. The fourth patient suffered an out-of-hospital cardiac arrest. His pacemaker, which had become necessary after valve replacement for infectious endocarditis, had registered an exit block. No further diagnostic assessment could be made as he died shortly after rehospitalization. Among patients presenting to the follow-up visit, no recurring infection was noted.

**Table 5:** Clinical outcome at the end of treatment visit (*n* = 74)

	*N* (% of patients)
Cured	49 (66)
Oral sequential antibiotic treatment ongoing	21 (28)
Treatment failure	0 (0)
Died	3 (4)
Other^2^	1 (1)

^1^Three patients (4%) were lost to follow-up (two of them because treatment ended during a hospital stay not related to the infection)

^2^One patient declined the prolongation of treatment which was recommended because of possible residual infection seen in imaging

## Discussion

The prospective data from the K-APAT cohort offer valuable insights into OPAT in Germany, shedding light on various aspects such as patient characteristics, type of infections, pathogens, complications, outcomes and parameters associated with the OPAT process in Germany.

One of the standout findings is the substantial reduction in inpatient days, amounting to 1782 days in 77 patients (mean 23 days). This not only underscores the growing need for OPAT but also emphasizes its potential to enhance the efficient utilization of healthcare resources. Recruitment was mainly from the university hospital, with fewer from other hospitals or physician offices. To some extent, this is expected because of the higher overall number of patients treated at the university hospital, and because at the tertiary care level the prevalence of infections requiring long treatment duration is likely higher. But it also seems to reflect that existing healthcare structures currently do not encourage OPAT and that the challenges might be even greater for non-university hospitals and non-university outpatient settings. Among these challenges are a lack of medical recommendations and structural factors, and that reimbursement by public healthcare insurance (which is mandatory for most patients in Germany) has not yet been regulated in detail.

As patient selection is crucial for safe and successful OPAT [1, 2], screening failures (*n* = 16/94, 17%) are expected and indicate a functional screening process emphasizing its critical role in ensuring the safety and success of OPAT. It is important to note that patients screened for this study had been previously evaluated by an ID physician as part of regular patient management, presenting a form of pre-screening which likely resulted in a certain pre-selection of patients more likely to be suitable for OPAT. Therefore, it can be assumed that an appropriate selection process results in even more screening failures. Previous studies demonstrated that ID specialist involvement improved treatment and reduced emergency room visits and readmissions [1, 15, 16]. Crucially, it also led to the conclusion that OPAT was unnecessary in 27% of patients, in which anti-infective treatment could be given orally or stopped [15].

As previously described [17, 18], approximately one-quarter of patients suffered from orthopaedic infection (*n* = 20, 26%). Other main entities treated included *S. aureus* bloodstream infection (*n* = 16, 21%) and infectious endocarditis (*n* = 11, 14%). Notably, the relatively high incidence of syphilis in Cologne (57.8/100.000 inhabitants in 2019) [19] explains the comparatively high proportion of patients with neurosyphilis (*n* = 9, 12%). Conversely, skin and soft tissue infections were underrepresented compared to international literature [20, 21], reflecting a comparatively low rate of methicillin-resistant *S. aureus* [22, 23] and that, in this study, only patients with at least 5 remaining days of IV treatment were included. Similar applies to urinary tract infections. Once OPAT is implemented on a large scale, it is likely that also patients requiring less than 5 days of OPAT can and will be offered OPAT, which will likely affect the type of patients and infections eligible for OPAT.

Flucloxacillin and penicillin G were the most frequently prescribed antimicrobials in our study. It has been discussed to use ceftriaxone instead of penicillin G in specific entities due to its extended dosing intervals, and it is noteworthy that in some cohorts, ceftriaxone was the most commonly used anti-infective substance [18, 20]. Our preference is to avoid the use of broad-spectrum agents if not necessary, and we found the required dosing intervals did not interfere with feasibility. This is an important finding and may be an argument for self-administered OPAT, as we do not think antibiotic stewardship principles should be compromised in OPAT. Regarding vascular access, PICC lines were the preferred choice for the majority of patients (*n* = 66, 87%). In spite of some advantages, midlines are not established in our setting and were therefore not employed [1, 24]. Fortunately, severe catheter-related complications were rare and did not lead to persisting symptoms, and no diagnosed catheter-related infections occurred. This outcome aligns with previous reports and confirms the safety and efficacy of OPAT with regard to vascular access [2, 17, 20, 24–26]. Adverse events that were not catheter-related were related to the infection or antimicrobials, not to their outpatient administration.

The rehospitalization rate of 21% before completion of OPAT compares to previously published data, reporting 3–26% [24, 27–29]. While a low rehospitalization rate can be considered indicative of effective patient selection, it is important to acknowledge that this rate is also influenced by the specific characteristics of the patients involved. In the context of this study, where haemato-oncological disease affects a rather large proportion of participants, the underlying medical conditions often require (planned or unplanned) readmission, contributing to the observed rate. Over half of the (planned and unplanned) rehospitalizations were due to the underlying disease or its treatment, the only rehospitalization clearly related to the infection treated by OPAT, the anti-infective used, or OPAT itself, was due to a (reversible) side effect of the anti-infective.

None of the four deaths was related to OPAT. The presence of severe malignancy in three out of the four deceased patients underscores the necessity for a comprehensive discussion to identify the individuals within this group who stand to benefit most from OPAT. This consideration allows them to enjoy the comfort of home, although it acknowledges the potential challenges associated with outpatient management in such cases.

This study offers the first comprehensive prospective cohort data on OPAT in Germany. Currently, OPAT does not play a large role in the German health system, emphasizing the need for local data to facilitate its implementation in the country. The study, conducted in a metropolitan region and mostly at a university hospital, may not fully represent other healthcare settings. Due to the large variety of patients, infections and their presentations, establishing a suitable control group is challenging and was not within the scope of this study. Moreover, it is essential to acknowledge that the COVID-19 pandemic had an impact on the recruitment process and the planned sample size was not reached. The sample size, although too small for the estimation of rare events like complications and outcomes, serves as a foundational dataset for future studies and the planning of OPAT implementation.

This study highlights the critical importance of patient selection. We suggest defining criteria guiding patient selection to avoid complications and unnecessary IV treatment. Furthermore, we emphasize the importance of closely adhering to existing recommendations that advocate involving an ID specialist [2]. In the lack of data guiding treatment monitoring, weekly clinical visits at the ID outpatient unit plus weekly home visits by care professionals appears to be a suitable approach. While there is insufficient evidence from previous studies to favour a certain model of OPAT administration [1], notably, the self-administration of anti-infectives using elastomeric pumps at home was demonstrated to be effective and reliable, with no relevant number of reported missed doses.

## Conclusion

In conclusion, this study underlines the feasibility and importance of implementing OPAT as an additional mode of treatment in Germany. However, structural factors such as dedicated staff, pathways of intersectoral and interdisciplinary communication, clear assignment of responsibilities, as well as the formulation of effective financing and regulations need to be established prior to a larger roll-out of OPAT in Germany.

### Supplementary Information

Below is the link to the electronic supplementary material.

Supplementary file1 (DOCX 18 KB)
