# Supplementary material for: Outpatient parenteral antimicrobial therapy (OPAT) in Germany: insights and clinical outcomes from the K-APAT cohort study
Source: Infection. 2024 Mar 13;52(4):1407–14. doi: 10.1007/s15010-024-02199-9 (PMC11289149; doi:10.1007/s15010-024-02199-9)
Supplement: Supplementary file 1 — Supplementary file1 (DOCX 18 KB) [file 15010_2024_2199_MOESM1_ESM.docx]

| **Patient:** | **Date:** | |
| --- | --- | --- |
| **Patient suitability** | **Yes** | **No** |
| Discharge possible (currently no surgical intervention required)   - No unclear clinical picture - No oral treatment option |  |  |
| Planned duration of treatment Minimum 5 days after discharge |  |  |
| Informed consent regarding treatment and catheter related risks |  |  |
| Informed consent regarding self-applied intravenous treatment (including dosing, duration and follow up visits |  |  |
| Patient able to come to the outpatient department twice weekly |  |  |
| Suitable intravenous access |  |  |
| If no: planned? |  |  |
| Patient trained in handling of IV line |  |  |
| *alternatively,* caregiver trained in handling the IV line |  |  |
| Informed consent signed |  |  |
| **Outpatient treatment** | **Yes** | **No** |
| Suitable living conditions |  |  |
| Wound care required |  |  |
| Patient instructed on how to proceed in case of complications regarding disease, catheter or treatment related complications |  |  |
| Patient received the information leaflet on how to proceed in case of complications |  |  |
| K-APAT patient documentation document explained and given to the patient |  |  |
| Patient contact data registered |  |  |
| General practitioner informed |  |  |
| **Appointments after discharge** | **Yes** | **No** |
| First delivery of medication and materials to the patient’s home:  Date: ____________________________ |  |  |
| First outpatient follow-up visit at the ID outpatient department (1. Week after discharge):  Date: ____________________________ |  |  |
| Follow up visit at another specialist department:  Datum: ____________________________ |  |  |
| **Physician** | | |
| Name | | |
| Signature | | |

**K-APAT Checklist (translated from German)**
